# Vitamin D Status Impacts Genital Mucosal Immunity and Markers of HIV-1 Susceptibility in Women

**DOI:** 10.3390/nu12103176

**Published:** 2020-10-17

**Authors:** Sharon M. Anderson, Andrea R. Thurman, Neelima Chandra, Suzanne S. Jackson, Susana Asin, Christiane Rollenhagen, Mimi Ghosh, Jason Daniels, Nikolas C. Vann, Meredith R. Clark, Gustavo F. Doncel

**Affiliations:** 1 CONRAD, Eastern Virginia Medical School, Norfolk, VA 23507, USA; ThurmaAR@evms.edu (A.R.T.); chandrn@evms.edu (N.C.); JacksoSS@evms.edu (S.S.J.); vannnc@evms.edu (N.C.V.); mclark@conrad.org (M.R.C.); DoncelGF@evms.edu (G.F.D.); 2 CONRAD, Eastern Virginia Medical School, Arlington, VA 22209, USA; 3 V.A. Medical Center, White River Junction, VT 05009, USA; Susana.N.Asin@Dartmouth.EDU (S.A.); Christiane.Rollenhagen@dartmouth.edu (C.R.); 4 Geisel School of Medicine at Dartmouth, Hanover, NH 03755, USA; 5 Milken Institute School of Public Health and Health Services, George Washington University, Washington, DC 20052, USA; mghosh@gwu.edu (M.G.); jasondaniels@email.gwu.edu (J.D.)

**Keywords:** vitamin D supplementation, HIV-1 susceptibility, immune regulation

## Abstract

While vitamin D insufficiency is known to impact a multitude of health outcomes, including HIV-1, little is known about the role of vitamin D-mediated immune regulation in the female reproductive tract (FRT). We performed a pilot clinical study of 20 women with circulating 25(OH)D levels <62.5 nmol/L. Participants were randomized into either weekly or daily high-dose oral vitamin D supplementation groups. In addition to serum vitamin D levels, genital mucosal endpoints, including soluble mediators, immune cell populations, gene expression, and ex vivo HIV-1 infection, were assessed. While systemic vitamin D levels showed a significant increase following supplementation, these changes translated into modest effects on the cervicovaginal factors studied. Paradoxically, post-supplementation vitamin D levels were decreased in cervicovaginal fluids. Given the strong correlation between vitamin D status and HIV-1 infection and the widespread nature of vitamin D deficiency, further understanding of the role of vitamin D immunoregulation in the female reproductive tract is important.

## 1. Introduction

Worldwide, it is estimated that over a billion people are at risk of vitamin D deficiency, and low vitamin D status has been linked to multiple disease states [1]. Current estimates suggest that nutritional interventions aimed at doubling the circulating levels of vitamin D are the most cost-effective way to reduce global mortality rates [2]. Vitamin D has long been known to play a role in modulating immune responses [3,4]. Macrophages, dendritic cells, and epithelial cells both respond to and produce 1,25(OH)_2_D, the biologically active form of vitamin D, as well express vitamin D receptors (VDRs) and CYP27B1, the enzyme that converts the circulating metabolite 25(OH)D into 1,25(OH)_2_D. Direct exposure to vitamin D suppresses immune activation, T-cell recruitment by chemokines and effector T-cell proliferation, while promoting the development of regulatory T cells (Tregs) [5,6,7,8]. The active form of vitamin D modulates the expression of multiple genes through a VDR response element, including the regulation of antimicrobial factors such as cathelicidin (LL-37), which are typically induced by toll-like receptor (TLR) activation [9,10,11]. 

Epidemiological data suggest an association between low serum 25(OH)D levels (used to define vitamin D status) and increased risk of adverse women’s reproductive health outcomes [12,13]. Vitamin D deficiency is prevalent in HIV-1-infected women, where upwards of 60% of individuals have levels lower than 50 nmol/L [14], and is associated with HIV disease progression and mortality [15,16]. A recent phase I clinical trial found enhanced antimicrobial activity in the plasma of HIV-infected individuals following one month of vitamin D supplementation [17]; however, little is known regarding vitamin D immune-regulation in the female reproductive tract (FRT), or how vitamin D status may impact mucosal HIV acquisition [18]. It has been proposed that vitamin D deficiency may predispose to an unbalanced pro-inflammatory response, including an increased production of pro-inflammatory cytokines [19,20,21], and decreased production of antimicrobial peptides, resulting in a lowered defense against vaginal infection. Vitamin D also promotes the differentiation and proliferation of keratinocytes in the epidermis [22], suggesting a role for the regulation of epithelial integrity in the genital tract. Long-term vitamin D supplementation has shown increased vaginal epithelial cell proliferation and VDR expression in rats [23,24] and post-menopausal women [25]. In premenopausal women, VDR expression in the vagina is also dependent on systemic vitamin D levels, as well as the phase of the menstrual cycle [26]. 

Based on the above evidence, we hypothesized that vitamin D supplementation of women with low levels of systemic 25(OH)D would result in increased antiviral activity and decreased susceptibility to ex vivo HIV infection in the female reproductive tract. In this proof-of-concept clinical study, the expression of markers of genital tract immunity and susceptibility to HIV-1 infection were evaluated in women receiving a high-dose oral supplementation (daily or weekly) of vitamin D for two months. We also investigated gene expression changes in cervicovaginal tissue to determine if vitamin D supplementation alters the expression of immune factors associated to antiviral and pro-inflammatory responses and HIV-1 infectivity.

## 2. Materials and Methods 

### 2.1. Clinical Study

Twenty women were enrolled in an outpatient open label study (CONRAD A14-131) that was approved by the Chesapeake Institutional Review Board (IRB#Pro00009891) and registered with ClinicalTrials.gov (NCT02186535). Participants were healthy, non-pregnant, HIV-negative women aged 21–50 years with regular menstrual cycles. At the time of enrollment, participants were eligible for the study if they had an insufficient vitamin D status, defined as serum 25(OH)D levels <62.5 nmol/L. Select exclusion criteria included: body mass index (BMI) >35 kg/m^2^, use of hormonal contraceptive methods or copper intrauterine device (IUD), currently breastfeeding, evidence of reproductive tract infections such as bacterial vaginosis (by Nugent score 7–10), yeast vaginitis, or sexually transmitted infections, risk factors for hypocalcemia or malabsorption syndromes, use of tobacco products, and systemic use within the past two weeks of non-steroidal anti-inflammatory drugs. All participants were required to refrain from vaginal intercourse 48 h prior to each genital sampling procedure.

Women were seen in 3 study visits and contacted by one scheduled follow-up safety telephone call after their final study visit. Volunteers were consented at visit 1 (V1) and underwent procedures to confirm their eligibility for the study. Baseline blood and genital samples were taken in the luteal phase (menstrual cycle days 18–26 ± 1 day) at visit 2 (V2, pre-supplementation). Participants were then randomized in a 1:1 manner and given a supplementation oral dosing regimen and their first dose of vitamin D—4000 IU daily or 50,000 IU weekly—in capsule form, which was then administered for 8 weeks. Participants were also given 1000 mg of calcium to take daily, and provided with sunscreen to use for the duration of the study. Two high-dose supplementation regimens were employed in order to ensure we achieved vitamin D serum concentrations higher than 75 nmol/L (30 ng/mL) following practice guidelines. As these conditions were met in both groups, and given the homogeneity of other data between the groups, the daily and weekly treatment groups were combined into pre- and post-treatment groups for subsequent analyses. Participants returned upon the completion of supplementation treatment for a repeat of blood and genital samples at visit 3 (V3, post-supplementation), also during the luteal phase.

### 2.2. Measurement of Serum and Cervicovaginal 25(OH)D 

25(OH)D concentrations in serum were evaluated at Lab Corp by an Immunochemiluminometric assay (ICMA). This assay was performed on the DiaSorin LIAISON^®^ instrument which measures both D2 and D3 together and reports a total 25-hydroxy vitamin D. The normal reference interval is 75−250 nmol/L (30−100 ng/mL).

Cervicovaginal fluid (obtained from cervicovaginal lavage (CVL) collection; see below) vitamin D levels were measured by the Endocrine Technologies Core (ETC) at the Oregon National Primate Research Center (ONPRC) using a Roche cobas e411 automatic clinical immunoassay platform (Roche Diagnostics, Indianapolis, IN). The analytical range of the vitamin D assay was 12.5−150 nmol/L (5.00–60.0 ng/mL). Samples were measured by the ETC in a single assay; the intra-assay coefficient of variation was 10.1%. The inter-assay coefficient of variation for the vitamin D assay in the ETC was 8.6%.

### 2.3. Cervicovaginal Lavage Collection 

Briefly, two 15 mL BD Falcon tubes were labeled for the collection and separation of CVL and CVL pellet. Using a syringe, 4 mL of normal saline was injected, lavaging the cervical fornices, and vaginal walls but not aimed directly at the cervical os and repeated approximately 4 times with the same fluid. Fluid was then aspirated carefully from the vagina and place in a labeled Falcon tube on ice. Within 30 min of collection, samples were centrifuged in a refrigerated centrifuge for 10 min at 800× *g*. Afterwards, the entire CVL supernatant was transferred to a second pre-labeled Falcon tube. The supernatants were then vortexed well before aliquoting into four cryovials and stored at −80 °C until evaluation.

### 2.4. Anti-HIV Activity of the Cervicovaginal Lavage 

Antimicrobial activity HIV-1 strain BaL was kindly provided by Dr. P. Gupta (University of Pittsburgh, PA, USA). Anti-HIV activity in CVL was determined using the TZM-bl indicator cell-line (AIDS reagent Repository, NIH), essentially as previously described [27]. Cells were seeded at 2 × 104 cells per well in a 96-well plate, and allowed to adhere overnight at 37° C. CVL samples were diluted 1:4 in TZM-bl media (phenol red-free DMEM (Invitrogen Life Technologies, Carlsbad, CA, USA) supplemented with 10% defined FBS (HyClone, Logan, UT, USA), 2 mM L-glutamine (Invitrogen Life Technologies), and 50 µg/mL Primocin (Invivogen, San Diego, CA, USA)) and incubated with a laboratory-adapted HIV-1 strain BaL at 250 tissue culture infectious dose (TCID50). The dose was selected to minimize virus-induced cytopathic effects, while maintaining the ability to measure a >1 log reduction in virus infectivity. Samples were incubated with the virus for 1 h at 37 °C and added to TZM-bl cells. Luciferase activity was measured after 48h upon the application of substrate beta-Glo (Promega, Madison, WI, USA). Uninfected cells and cells treated with CVL alone were used to determine background luminescence expressed as relative light units (RLUs). All conditions were tested in triplicates and repeated twice. To calculate the percentage inhibition, RLU values of “virus only” wells were averaged and set to 100%. The viability of cells upon treatment with CVL was quantified using the CellTiter 96^®^ AQueous One Solution Cell Proliferation Assay (Promega) according to the manufacturer’s instructions. Briefly, the reagent was added directly to cell cultures and incubated for 30 min at 37 °C followed by reading the plate in a plate reader at optical density 490 nm.

### 2.5. Soluble Mucosal Proteins in CV Secretions

Total protein concentrations in CVL specimens harvested and stored as describe above were quantified by a microBCA assay. CVL specimens were diluted to enable interpolation from a standard curve. Concentrations below the lower limit of detection (LLOD) were set to the midpoint between 0 and the LLOD and corrected for dilution factor. Mucosal protein concentrations were normalized to the total protein content and expressed as mg/mL of CVL. CVL samples were stored at −80 °C until shipped via overnight courier to the laboratory of Dr. Mimi Ghosh (George Washington University, Washington, DC, USA) and assayed for soluble antimicrobials, secretory leukocyte protease inhibitor (SLPI), Elafin, Human beta defensin 2 (HBD2), and LL-37 by ELISA. SLPI and Elafin ELISA kits were purchased from R&D Systems (Minneapolis, MN, USA), HBD2 from PeproTech (Rocky Hill, NJ, USA) and LL-37 from Hycult Biotech (Wayne, PA, USA). All assays were performed according to the manufacturer’s protocols. The levels of proteins were quantified based on standard curves obtained using a Microplate Reader (Biotek, Winooski, VT, USA). Concentrations below the lower limit of detection (LLOD) were set to the midpoint between 0 and the LLOD and corrected for dilution.

### 2.6. Analysis of Vaginal Immune Cell Populations and Histology

At baseline and post-supplementation, two vaginal biopsies were obtained from each participant for histology and immune cell phenotype analysis by immunohistochemistry (IHC). One tissue was paraffin-embedded following fixation in formalin, and one was cryopreserved. 

For the formalin-fixed tissues, one vaginal biopsy from each visit was placed in 10% neutral buffered formalin for 24–48 h (hrs), transferred to an embedding cassette, and processed [28]. The antigens were detected using an AEC chromogen–substrate kit (SkyTek Labs, Mississauga, ON, Canada) and mounted with Accergyl mounting media (Accurate Chemicals, Westbury, NY, USA). The cell phenotype was identified using specific monoclonal antibodies against CD45 (NCL-L-LCA, Leica Biosystems, Buffalo Grove, IL, USA), CD3 (NCL-L-CD3-565, Leica Biosystems), CD8 (NCL-L-CD8-295, Leica Biosystems), CD1a (C137757, LSBio, Seattle, WA, USA), CCR5 (MAB181, R&D Systems, Minneapolis, MN, USA), and CD4 (M7310, DAKO, Santa Clara, CA, USA). Positive stained cells were counted in 5–6 random fields/tissue under the microscope (Nikon E-800). Cell density was expressed as cells/mm^2^. 

For the tissues undergoing cryopreservation, one vaginal biopsy from each visit was placed in an empty cryovial on ice and then embedded in Optimal Cutting Temperature (O.C.T.) compound (Tissue-Tek, Torrance, CA, USA) at −20 °C. The blocks were kept frozen (−20) until used. In total, 5−6-micron sections were cut in the cryostat microtome (Tissue-Tek) and processed for immunohistochemistry (IHC) staining for CD4 and CCR5. In brief, the slides were brought to room temperature and washed with Phosphate buffered saline (PBS). Sections were then fixed in cold acetone for 15 min washed with PBS and then incubated for 30 min with specific serum protein to block non-specific binding. The tissues were then incubated with CD4 (1:20, DAKO) or CCR5 (1:100, R&D) at 4 °C overnight. The remaining processing and analysis were completed as described above for paraffin-embedded sections.

Epithelial layers and thickness were analyzed on Hematoxylin and Eosin (H&E)-stained slides under a microscope inserted with a reticle, and expressed as numbers for layers and micrometers for thickness [28].

### 2.7. HIV-1 p24 Antigen Production from Ectocervical Tissues after Ex Vivo HIV-1 Infection

Tissue biopsies were placed in a microcentrifuge tube containing complete Leibovitz 15 (cL15) tissue culture media (Gibco, Grand Island, NY, USA) supplemented with 10% heat-inactivate Fetal Bovine Serum (Hyclone, Logan, UT, USA), 100 units/mL Penicillin and 100 µg/mL Streptomycin (Gibco). Explants and biopsies were kept on ice and shipped via overnight courier to the laboratory of Dr. Susana Asin (V.A. Medical Center, White River Junction, VT, USA). The following day, the biopsy was stabilized for 4 h at 37 °C and infected with 10^4^ TCID_50_ of HIV-1_BaL_ in a final volume of 100 µL per well, in a 96-well plate and processed as previously described [29]. 

### 2.8. RNA Isolation

One vaginal biopsy from each visit was placed in an RNA later solution (Ambion AM7021; Invitrogen Life Sciences, Waltham, MA, USA) and frozen at −80 °C. For RNA isolation, tissues were homogenized with TRIzol (Invitrogen Life Technologies) using OMNI international homogenizer and total RNA was extracted and then purified using RNeasy mini kit columns (Qiagen, Germantown, MD, USA) according to the manufacturer’s instructions.

### 2.9. Quantitative Real-Time PCR (qPCR)

Quantitative real-time PCR (qPCR) was performed as previously described [30]. Briefly, PCR was performed on a Roche LightCycler Carousel-based system using LightCycler FastStart DNA Master SYBR Green I (Roche, Indianapolis, IN, USA) according to the manufacturer’s instructions. All primers were purchased from Qiagen with catalog numbers as follows: Vitamin D Receptor (Catalog # 330001 PPH02123F), ALOX12 (Catalog # 330001 PPH05791C), ISG15 (Catalog # 330001 PPH01333F), RSAD2 (Catalog # 330001 PPH08142A), IL8 (Catalog # 330001 PPH00568A), FLG (Catalog # 330001 PPH24283A), CCL8 (Catalog # 330001 PPH01167B), CXCL11 (Catalog # 330001 PPH00506A), RPTN (Catalog # 330001 PPH18382A).

### 2.10. Statistical Analysis

Statistical analyses were performed with either SAS version 9.3 (Cary, NC, USA) or GraphPad Prism version 8.2 (San Diego, NC, USA). Descriptive statistics included mean, median, and standard deviation for continuous variables and frequencies and percentages for categorical variables. Continuous endpoints from supplementation cohorts were compared using an independent samples t test for normally distributed data or Wilcoxon–Mann–Whitney test for non-normally distributed data. For categorical variables, the chi square statistic or Fisher’s exact tests were used as indicated by expected cell size. Paired comparisons using a paired t test or Wilcoxon signed rank sum test were performed on different variables to compare pre- versus post-supplementation samples. To achieve normality, data were log transformed for the p24 production, soluble mucosal proteins and immune cell types variables [31,32]. For the analyses of p24 antigen production, the categorization of infected versus not infected samples was conducted using 0.1 log steps and a Boolean method, followed by a comparison of infected versus not infected samples by Fisher’s exact test or McNemar’s test as appropriate. Statistical significance was determined at the level of alpha = 0.05. Fold changes for qPCR data were evaluated by the relative change in gene expression using the 2-ΔΔCt method and transformed into positive and negative folds by fractional fold changes using inverse transformation [33,34]. Statistical significance was determined to be met with fold changes of a magnitude greater than 2 (i.e., >2 or <−2). 

## 3. Results

### 3.1. Demographic Data

A total of 54 women were screened for the study, and 23 enrolled. Of the 23 women enrolled, 20 women (10 each in the daily and weekly supplementation arms of the trial) completed all study visits, and represent the analysis population (Table 1). There were no differences between the weekly and daily oral supplementation groups in any of the variables analyzed except for BMI, which was significantly higher for women in the weekly supplementation arm than daily supplementation arm. However, this difference is likely not clinically significant, as the BMI for both groups fell well within the national average, and split the overall median [35]. 

### 3.2. Vitamin D Levels Post-Supplementation

**Figure 1:**
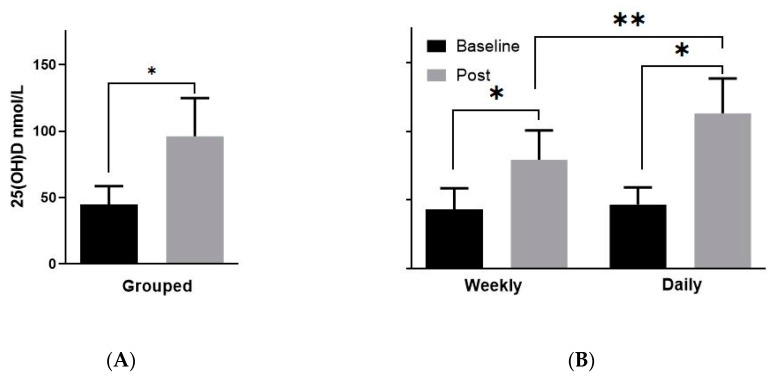
Vitamin D supplementation increased mean serum 25(OH)D concentration to above adequate levels >75 nmol/L (30 ng/mL). Women were sampled before and after 8 weeks of either weekly (*n* = 10) or daily (*n* = 10) high-dose vitamin D. (**A**) Grouped mean (± SD) serum 25(OH)D concentrations from both weekly and daily arms (*n* = 20) before and after vitamin D supplementation. (**B**) Mean (± SD) serum 25(OH)D concentrations from weekly and daily supplementation arms. * Denotes significant difference between baseline and treatment; ** denotes significant difference between daily and weekly supplementation groups following treatment.

At the baseline visit prior to supplementation, the mean serum 25(OH)D concentration of the analysis population was 45.9 ± 13.7 nmol/L, which is defined as vitamin D deficiency per the Endocrine Society’s 2011 clinical practice guidelines [1]. As expected, following two months of high-dose oral vitamin D repletion regimens with 4000 IU daily or 50,000 IU weekly, the overall mean systemic levels of 25(OH)D significantly increased to the adequate range (96.2 ± 28.7 nmol/L, *p* < 0.0001, Figure 1A). When broken down by the supplementation regimen, the same pattern was observed following daily (46.4 ± 4.0 versus 113.1 ± 8.0 nmol/L, *p* < 0.0001, Figure 1B) and weekly (43.2 ± 4.7 versus 79.4 ± 6.7 nmol/L; *p* = 0.0004, Figure 1B) supplementation. The post-supplementation 25(OH)D levels did differ significantly between the two treatments (*p* = 0.0048), with the daily group displaying higher circulating 25(OH)D as compared to the weekly supplementation group. However, given that both groups demonstrated improvement to the adequate range of serum 25(OH)D levels based on guidelines, and the homogeneity of all other data between the groups, we have combined the daily and weekly treatment groups into one pre- and post-treatment group for subsequent analyses in order to maximize the sample size and statistical power.

Additionally, we also quantified the levels of 25(OH)D in cervicovaginal fluid (CVF) from the CVL samples as an exploratory biomarker of vitamin D uptake and status. To our knowledge, 25(OH)D levels have not previously been evaluated in CVF, and while there are no established benchmarks in this matrix, we found that, in contrast to serum levels, the CVF 25(OH)D levels, quantified using LC MS/MS, decreased from pre-supplementation (80.0 ± 9.0 nmol/L) to post-supplementation (70.9 ± 10.0 nmol/L, *p* = 0.0014) when both weekly and daily cohorts were combined.

### 3.3. Vitamin D Supplementation Alters Cervicovaginal Immune Cell Expression 

Given the role of vitamin D in systemic immunoregulation processes, we next investigated the density of immune cells and phenotype within vaginal tissue biopsies following supplementation. An anatomical quantification of tissue biopsies revealed a significant change in epithelial thickness due to vitamin D supplementation (pre: 225.5 ± 55.1 versus post: 265.7 ± 88.0, *p* = 0.05) (Table 2). However, this increase was likely not due to an increase in the number of cell layers as we found no significant difference between pre-supplementation (20.8 ± 3.0) and post-supplementation (21.3 ± 3.1, *p* = 0.50) in the number of cell layers within the epithelium. 

We noted no significant changes across most phenotypic immune cell markers in the epithelium or lamina propria (LP) (CD45, CD3, CD8, CD1a, and CCR5) between pre- and post-supplementation (Table 2). However, CD4+ cell expression decreased significantly in both the LP (pre: 121.9 ± 42.9 versus post: 92.2 ± 33.1, *p* = 0.02), and the epithelium (61.9 ± 40.3 versus post: 44.2 ± 24.2, *p* = 0.04).

### 3.4. Vitamin D Supplementation Effects on Gene Expression and Secretion of Innate Immune Factors 

Female reproductive tract secretions contain a wide array of innate immune factors with broad-spectrum inhibitory activity against pathogens, including HIV [36]. We characterized the levels of elafin, HBD-2, LL-37, and SLPI in CVL collected from women at pre- and post-supplementation visits. However, we found no significant changes in the secretion of any of the antimicrobial peptides (AMPs) in CVL samples between pre- and post-supplementation (Table 3). 

**Figure 2:**
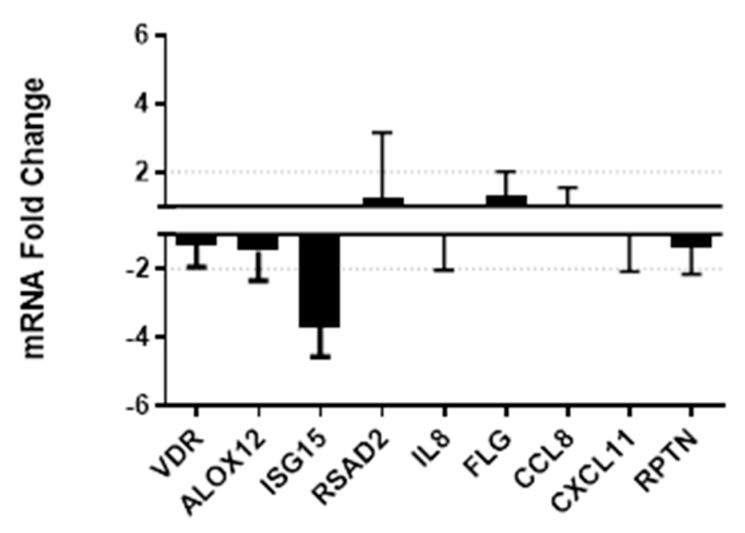
Quantitative real-time PCR was performed on RNA isolated from vaginal biopsies taken at V2 baseline versus V3 post-supplementation. The relative changes in gene expression were calculated using the 2-ΔΔCt method, and expressed as positive or negative fold change compared to V2 with 1 or -1 as reference points, respectively.

A discriminatory panel of genes previously identified by our group as signals of mucosal inflammation and immune activation [30,37], as well as genes known to be regulated by vitamin D status systemically, were selected for the qPCR assessment in vaginal tissue samples including: VDR, ALOX12, ISG15, RSAD2, IL8, FLG, CCL8, CXCL11, RPTN. Using qPCR we found a 3.7-fold decrease in interferon-stimulated gene 15 (ISG15; Figure 2) which has been shown to block retrovirus release and inhibit HIV-1 replication [38,39]. However it has also been suggested as a potential marker of chronic immune activation in HIV-1-infected individuals. [40]. We found no other significant changes in gene expression levels for genes of interest (Figure 2). 

### 3.5. Inhibition of HIV-1 by CVL Fluid Following Vitamin D Supplementation 

We obtained CV fluid from women during pre- and post-supplementation visits to determine whether CVL post-vitamin D supplementation would reduce HIV infectivity in target cells. HIV inhibition activity of fluids was not significantly different between pre- and post-supplementation (Table 4). 

### 3.6. Vitamin D Supplementation and Susceptibility of Cervical Tissues to Ex Vivo HIV Infection 

Cervical tissue biopsies were taken at baseline and post-supplementation and shipped overnight in medium to Dr. Asin’s lab. Approximately 24 h after collection, tissues were challenged with HIV-1 BaL and cultured for 21 days. HIV p24 antigen production was measured by different approaches (e.g., day 21, area under the curve (AUC), cumulative, maximum (Max), or soft endpoint (Soft)) [31] in pre- and post-supplementation tissues (Table 5). Although p24 levels were lower after vitamin D supplementation, none of these differences achieved statistical significance due the variability of the data. Moreover, we found no correlation between serum 25(OH)D and p24 antigen production (data not shown).

## 4. Discussion

This exploratory clinical trial is the first to assess the impact of vitamin D supplementation on CVF and tissue markers of antimicrobial and anti-inflammatory activity associated with mucosal susceptibility to HIV-1 infection, as well as to directly measure whether oral supplementation could reduce HIV-1 infectivity in either cells exposed to CV fluid or tissue. As expected, both weekly and daily vitamin D supplementation regimens improved serum 25(OH)D concentrations in deficient women to above the adequate range. In contrast to serum values, 25(OH)D levels measured in CVL fluid decreased between pre- and post-supplementation. This disparity may explain the lack of findings in FRT factors, and indicates that oral supplementation of vitamin D may not be sufficient to alter cervicovaginal levels, or may result in different kinetics in local compartments. While 25(OH)D has been reported in other body fluids, including cerebrospinal and follicular fluid [41,42], we are not aware of previous assessments in cervicovaginal fluid, and therefore no benchmark criteria exist for standard 25(OH)D levels in this biological matrix. In addition, there was no correlation between the CVL and plasma levels of vitamin D, nor any correlation between CVL 25(OH)D levels and reported experimental endpoints. Our data show that the concentration of vitamin D in the CVF was higher at baseline as compared with plasma levels, but lower after supplementation.

Two salient changes from baseline were a reduction in mucosal CD4+ cells and ISG15 gene expression. ISG15 is an ubiquitin-like protein with antiviral functions against numerous viruses, including HIV-1 [43,44], ISG15 has been shown to correlate with HIV-1’s viral load and CD4+ expression in HIV-infected individuals [40,45], and can be regulated by 1,25(OH)_2_D_3_ in vitro [43,46]. When released extracellularly, secreted ISG15 can function as a cytokine and mediate antiviral immune responses, including promoting dendritic cell maturation [47], releasing IFNγ from T cells [48] and subsequently increasing T-cell activation, inducing anti-HIV activity of macrophages as part of a complement of exosomally released factors [49], and increasing chemotaxis [50]. Taken as a whole, the downregulation of ISG15 could result in a weakened protective response against HIV-1 infection. While we did not observe any significant differences in p24 infection, it is possible that lower levels of ISG15 activity resulted in reduced chemoattraction and stimulation of CD4+ cells. The resulting decrease in the pool of target cells may have led to the trend towards lower virus replication that we observed, although not enough to reach statistical significance. 

In spite of significant decreases in HIV-1 target immune cell populations expressing the HIV-1 receptor CD4, and a nearly 4-fold decrease in the key antiviral gene ISG15, providing evidence that systemic vitamin D can impact the FRT immune system, these results did not translate into significant changes in antimicrobial protein levels in CVL secretions or HIV p24 production in ex vivo tissues. Although vitamin D supplementation achieved adequate values in the systemic compartment, the paradoxical lower level of vitamin D in CVF may have prevented significant changes in the FRT. Topical CV administration of vitamin D may be needed in order to achieve these changes. Further elucidation of the proper dose, route, and/or form of vitamin D supplementation to increase FRT 25(OH)D levels is needed to determine whether the hypothesized vaginal health changes and outcomes, including greater prevention against mucosal susceptibility to HIV-1 infection, are directly associated with elevated vitamin D concentrations. 

Along this line, we noted a decrease in CD4+ cells in both the lamina propria (subepitheial connective tissue) and epithelium of the CV mucosa after two months of oral supplementation. An immune cell response has not been previously shown in genital mucosal samples, but these findings are in agreement with data from peripheral blood immune cell populations, where treatment with vitamin D compounds results in decreased frequencies of infected CD4+ T cells, and the preferential downregulation of activated T cells expressing HLA-DR [51,52]. The immunohistochemical analysis also demonstrated a significant increase in epithelial thickness in vaginal tissue samples when women transitioned from an insufficient to sufficient vitamin D status. These results are consistent with animal data showing that vitamin D-deficient mice have reduced epithelial barrier functions and intestinal microbiome, resulting in a greater susceptibility to infectious pathogens [53].

The production of p24 antigen from tissue biopsies or explants after ex vivo infection by HIV-1_BaL_ has been used as a model for early mucosal HIV infection in women, and as an exploratory safety endpoint in phase I HIV prevention studies [32,54,55]. While there are reports of enhanced HIV-1 replication by the vitamin D metabolites [56,57,58], most in vitro studies suggest that vitamin D plays a protective role against HIV-1 infection in immune cells. In peripheral blood mononuclear cells (PBMCs) isolated from healthy donors and exposed to physiological ranges of active vitamin D, there was significant decrease in p24 levels, and p24-infected CD4+ cells, correlating with the upregulation of VDR-regulated antiviral factors [51]. In a Cape Town-based study, p24 production from participant PBMCs was attenuated following 6 weeks of oral vitamin D3 supplementation; however, mucosal samples were not evaluated in this cohort [59]. In our study, we did not observe significant differences in HIV p24 antigens at any time points tested. It is worth noting that shipping tissues overnight prior to infection might have altered the tissues, masking intrinsic differences in HIV-1 infection responses due to vitamin D status. 

Elafin, LL-37, SLPI, and HβD are all cationic antimicrobial polypeptides (AMPs), a group of molecules which are considered as the most important effector molecules of mucosal innate immunity against bacterial and viral pathogens [60]. Although the antimicrobial factors assessed in the present study are known to have anti-HIV-1 activity, and serve as biomarkers of FRT immune cell function [27,61], we did not find a change in expression of elafin, HβD, SLPI or LL-37 in cervical vaginal fluid post-vitamin D supplementation. Antimicrobial factors such as LL-37 are induced by toll-like receptor (TLR) activation, and are regulated by the active form of vitamin D due to a VDR response element [9]. When serum vitamin D is insufficient, the ability to produce LL-37 is impaired [10,11]. Thus, one potential mechanism for vitamin D’s impact on innate immunity against HIV infection is via TLR-induced inhibition of HIV replication, which is dependent on functional vitamin D pathways [56]. In vitro studies of peripheral PMBCs using both circulating and active forms of vitamin D have demonstrated an increased expression of antiviral genes, including elafin and cathelicidin [51,52]. However, in these studies, the innate vitamin D status of donors was not documented, and gene expression was dependent on exogenous treatment with calcidiol or calcitriol at doses mirroring physiological concentrations. 

Using qPCR, we evaluated a panel of genes selected for their role in vitamin D and immune response pathways; however, other than ISG15, no significant changes in gene expression were noted. While VDR gene expression has previously been shown to correlate with plasma vitamin D status and antiviral factors in genital mucosa [5] in healthy participants, we saw no changes in CV mucosal VDR expression or correlation with antiviral activity (data not shown). Furthermore, although we did not see a significant change in VDR expression, the expression of VDR can be regulated by other factors outside of vitamin D. Menstrual phase has been shown to have some effect on VDR expression, and a quantifiable but non-significant negative link between serum 25(OH)D and vaginal VDR expression has been reported [26]. While we cannot rule out the potential impact of the menstrual cycle on VDR expression because we did not measure serum hormone levels during this study, pre- and post-supplementation visits were both scheduled during the luteal phase to lessen the impact of hormone levels, and therefore on changes in VDR, or other key factors expression. Our CVL data also suggest that plasma and CV levels of 25(OH)D are not correlated, and systemic supplementation did not result in higher 25(OH)D levels in the CVF. Taken together these data suggest that local rather than systemic vitamin D may be a more important factor in immune regulation within the FRT.

Currently, there is no standard vitamin D repletion regimen for adults, nor consensus on optimal values for sufficient status. While there are conflicting recommendations between the Institutes of Medicine (IOM) and the Endocrine Society regarding daily intakes and target serum levels, we elected to adhere more closely to the Endocrine Society’s guidelines, as they take into consideration a wider array of health benefits associated with vitamin D. The supplementation dosage and duration we chose was based on studies showing that after 8 weeks of treatment with 50,000 IU weekly, the mean serum levels of patients increased from 48 to 92.9 nmol/L [62]. On this treatment regimen, vitamin D deficiency was corrected (levels raised to >75 nmol/L) in 50–60% of cases [63,64]. Additionally, several dosing studies have determined 4000 IU to be optimal dosage to maintain serum levels above 75 nmol/L (30 ng/mL), with target serum levels reached within 3 months following the start of supplementation [65,66]. Our study duration was 8 weeks, as this timeframe has been demonstrated to adequately supplement vitamin D, and to ensure proper adherence to daily and weekly vitamin D regimens without loss to follow up. 

Body weight is widely acknowledged to be one of many health factors that can impact vitamin D status [67]. As vitamin D is a lipid-soluble molecule, body fat can act as a reservoir for storage, ultimately decreasing the bioavailability of vitamin D as body mass index (BMI) increases. Despite randomization, there was a significant difference between the mean BMI for the daily (24.99 kg/m^2^) as compared to the weekly (29.83 kg/m^2^) oral repletion groups. Both study arms fell within the overweight, not obese, category, and within the national average BMI for women in the United States, 29.6 [35]. Furthermore, while BMI negatively correlates with serum 25(OH)D in both men and women, the greatest impact to vitamin D deficiency is seen in individuals with BMI >30 [68], above the mean levels of either cohort evaluated here. However, we cannot rule out that this population difference in average BMI affected weekly versus daily outcomes. Data comparisons between weekly and daily supplementation showed no significant difference in gene or protein expression, the inhibition of HIV-1, or any other measured outcome besides serum 25(OH)D and the data sets were largely homogenous. 

Here we present the first investigation of the impact of oral vitamin D supplementation on markers of immune response and HIV-1 infectivity of CV tissues conducted in women. Our data, in contrast to in vitro data [7,20,51,57,58], suggest that increasing systemic vitamin D levels may have a limited impact on HIV-1 protection of the CV mucosa. However, this interpretation does not support large-scale epidemiological studies that show that vitamin D deficiency is one of the most common comorbidities associated with HIV infection and that HIV-infected individuals show attenuated HIV-1 replication with vitamin D supplementation [14,16,17,18,59]. One possibility is that vitamin D when supplemented orally provides protection against HIV mostly at the systemic level. This difference in biodistribution could explain the disparity in the results from mucosal and clinical outcomes. The vast body of in vitro evidence for the potential protective benefits of vitamin D against HIV in the CV mucosa suggests a follow-up study is warranted to investigate the impact of vaginally applied vitamin D3 on mucosal markers and HIV-1 susceptibility. Given the strong correlation between vitamin D status and HIV-1 infection and the widespread nature of vitamin D deficiency, especially in areas where HIV-1 infections are most prevalent, it is imperative to understand the biological basis for any potential impacts vitamin D has on immune function. 

## Figures and Tables

**Table 1:** Demographics of analysis population.

Variables	Total	Daily	Weekly	*p*-Value (Daily Versus Weekly)
Analysis Population	20	10	10	
Average Age (years) ^1^	37.05 ± 6.33	37.20 ± 1.97	36.90 ± 2.14	0.92
Average BMI (kg/m^2^) ^1^	27.41 ± 4.57	24.99 ± 1.25	29.83 ± 1.24	0.01
Education (years) ^1^	14.93 ± 2.21	14.80 ± 2.35	15.05 ± 2.19	0.80
Gravidity ^1^	2.45 ± 1.50	2.30 ± 1.49	2.60 ± 1.58	0.67
Parity ^1^	1.90 ± 1.29	2.0 ± 1.25	1.8 ± 1.4	0.74
Vaginal pH at V1 ^1^	4.12 ± 0.31	4.10 ± 0.32	4.14 ± 0.33	0.78
Vaginal Nugent Score at V1 ^1^	1.80 ± 1.70	1.40 ± 1.65	2.20 ± 1.75	0.31
**Race and Ethnicity**				
Hispanic Ethnicity	4	3	1	-
Non-Hispanic Black	7	1	6	-
Non-Hispanic White	8	5	3	-
Asian	1	1	0	-

^1^ All values expressed as group mean ± standard deviation.

**Table 2:** Changes in vaginal immune cell expression levels due to vitamin D supplementation.

Variables	Pre-Supplementation ^1^	Post-Supplementation ^1^	*p*-Value
Epithelial Thickness (µm)	225.5 ± 55.1	265.7 ± 88.0	0.05
Number of Cell Layers	20.8 ± 3.0	21.3 ± 3.1	0.5
Vaginal immune cell populations/phenotype (cells/mm^2^)			
CD45 Epi	120.5 ± 43.0	117.1 ± 52.9	0.8
CD45 LP	95.9 ± 37.1	109.0 ± 44.7	0.2
CD3 Epi	93.5 ± 41.4	93.1 ± 47.0	0.9
CD3 LP	68.9 ± 32.7	79.6 ± 38.0	0.3
CD8 Epi	78.3 ± 37.6	68.8 ± 43.9	0.08
CD8 LP	44.5 ± 26.1	49.1 ± 26.6	0.7
CD1a Epi	45.0 ± 17.9	43.5 ± 20.4	0.7
CD1a LP	0.1 ± 0.6	1.4 ± 2.7	0.09
CD4 Epi	61.9 ± 40.3	44.2 ± 24.2	0.04
CD4 LP	121.9 ± 42.9	92.2 ± 33.1	0.02
CCR5 Epi	0.0 ± 0.0	0.3 ± 0.9	0.2
CCR5 LP	25.1 ± 11.2	24.2 ± 9.7	0.7

^1^*n* = 20 pre- and post-supplementation groups.

**Table 3:** Antiviral protein levels in cervicovaginal fluid pre- and post-vitamin D supplementation.

Mediator (pg/mL)	Pre-Supplementation ^1^	Post-Supplementation ^1^	*p*-Value
Elafin	2401.2 ± 2168.8	1899.3 ± 1765.8	0.20
HBD-2	39.3 ± 69.7	23.0 ± 28.4	0.26
LL-37	29,459.0 ± 49,726.2	18,184.4 ± 3340.2	0.18
SLPI	581.7 ± 700.0	453.5 ± 481.1	0.54

^1^*n* = 20 pre- and post-supplementation groups.

**Table 4:** Inhibition of HIV-1 by cervicovaginal lavage (CVL) fluids at pre- versus post-vitamin D supplementation.

Treatment Group	% Inhibition Pre-Supplementation	% Inhibition Post-Supplementation	*p*-Value
Overall (*n* = 19)	69.68 ± 40.67	59.60 ± 33.67	0.15
Weekly (*n* = 9)	83.81 ± 21.14	66.86 ± 26.61	0.05
Daily (*n* = 10)	56.97 ± 50.32	53.06 ± 39.22	0.66

**Table 5:** HIV replication in cervical biopsies at pre- versus post-vitamin D supplementation.

Variable ^1^	Pre-Supplementation ^2^	Post-Supplementation ^2^	*p*-Value
P24_Day 21	192.03 ± 305.88	88.71 ± 159.39	0.12
P24_Soft	194.4 ± 304.65	98.43 ± 159.44	0.36
P24_Maximum	198.32 ± 302.3	109.93 ± 162.47	0.22
P24_Cumulative	240.9 ± 336.49	168.69 ± 204.94	0.30
P24_AUC	1012.47 ± 1294.33	868.64 ± 971.37	0.52

^1^ HIV p24 antigen production is expressed as pg/mL · mg tissue. ^2^ *n* = 20 pre- and post-supplementation groups (Mean ± SD).
